# Latent profiles of compassion fatigue and their association with resilience and turnover intention among nurses caring for patients with gastric cancer: a cross-sectional study

**DOI:** 10.3389/fpubh.2026.1799278

**Published:** 2026-04-24

**Authors:** Jinjin Du, Hui Hou

**Affiliations:** Department of General Surgery, The First Affiliated Hospital with Nanjing Medical University, Nanjing, China

**Keywords:** compassion fatigue, gastric cancer, latent profile analysis, night shifts, nurses, resilience

## Abstract

**Background:**

Oncology nurses caring for patients with gastric cancer are exposed to high-acuity workloads and sustained patient suffering, which may increase compassion fatigue. Prior research has largely examined average relationships across the full sample, which can obscure meaningful differences between nurses. This study aimed to identify latent profiles of compassion fatigue and examine the protective role of psychological resilience and the influence of work characteristics on profile membership.

**Methods:**

A cross-sectional sample of 678 nurses from tertiary hospitals who provided care for patients with gastric cancer was recruited. Latent profile analysis (LPA) was conducted using the three Professional Quality of Life (ProQOL) dimensions: Compassion Satisfaction, Burnout, and Secondary Traumatic Stress. Psychological resilience was assessed using the Connor–Davidson Resilience Scale (CD-RISC). Multinomial logistic regression was applied to determine predictors of profile membership.

**Results:**

Three profiles were identified: Thriving (22.0%; high compassion satisfaction with low burnout and secondary traumatic stress), At-risk (47.9%; moderate levels across dimensions), and Distressed (30.1%; low compassion satisfaction with high burnout and secondary traumatic stress). Turnover intention differed substantially across profiles: 80.4% of nurses in the Distressed profile reported high turnover intention compared with 6.0% in the Thriving profile. Psychological resilience was independently associated with a greater likelihood of Thriving membership relative to Distressed membership (OR = 1.18, *p* < 0.001). Frequent night shifts (>5/month) were associated with reduced odds of Thriving membership (OR = 0.25, *p* < 0.001), whereas receipt of psychological training was associated with increased odds of Thriving membership (OR = 2.10, *p* = 0.009).

**Conclusion:**

Nearly one-third of nurses caring for patients with gastric cancer experienced severe compassion fatigue accompanied by high turnover intention. Psychological resilience appears protective, and modifiable work characteristics—particularly night-shift burden and access to psychological training—are meaningfully associated with risk status.

## 1 Introduction

Nurses caring for patients with gastric cancer constitute a critical workforce managing complex perioperative care and nutritional rehabilitation, yet they face intense exposure to patient suffering and prolonged night shifts (1, 2). Compared with broader oncology nursing populations, this subspecialty may face a distinctive combination of perioperative management, nutritional rehabilitation, and sustained symptom burden. This environment heightens vulnerability to occupational stress, often resulting in burnout and secondary traumatic stress, which coexist paradoxically with compassion satisfaction—a dynamic captured by the Professional Quality of Life (ProQOL) framework (3, 4). While burnout is linked to adverse outcomes like medical errors and diminished patient safety (5), most existing oncology research examines average patterns across samples that often aggregate diverse tumor types. This may obscure clinically important differences between nurses, including groups who remain well and those who experience marked distress despite working in the same environment (6, 7).

Psychological resilience is increasingly recognized as a protective capital that buffers job demands, showing consistent inverse associations with emotional exhaustion across nursing specialties (8, 9). Conversely, structural stressors, particularly circadian-disrupting night shift schedules, are robust predictors of mood disturbances and poor recovery (10, 11). Although studies have linked resilience and shift work to ProQOL outcomes (12, 13), no research has yet applied person-centered Latent Profile Analysis (LPA) to characterize how these factors interact to shape distinct adaptation patterns specifically within gastric cancer nursing.

To address this gap, this study utilized LPA to identify unobserved subgroups of gastric cancer nurses characterized by distinct configurations of compassion satisfaction, burnout, and secondary traumatic stress. We examined the external validity of these profiles in relation to turnover intention, job satisfaction, and work engagement. Crucially, the study sought to determine whether resilience acts as an independent protective factor increasing the likelihood of belonging to “thriving” profiles, and whether high monthly night shift frequency and insufficient psychological training predict membership in “distressed” profiles. By integrating individual and environmental determinants, this research aims to provide a nuanced empirical basis for stratified interventions to sustain this specialized workforce (5–7).

## 2 Methods

### 2.1 Study design and setting

This was a multicentre, cross sectional questionnaire survey targeting gastric cancer nurses working in tertiary hospitals in China. The study aimed to identify latent profiles of compassion fatigue and to examine the role of resilience and work related factors in profile membership and occupational outcomes among this specific subspecialty workforce. The reporting followed common methodological standards for observational studies in nursing research (14).

### 2.2 Participants and sampling

This study was conducted in Tertiary Grade A hospitals across five economically developed and medically advanced provinces and municipalities in Eastern and Southwestern China, specifically Sichuan, Zhejiang, Jiangsu, Shanghai, and Fujian.

Eligible participants were registered nurses who: (a) held a valid nurse practice license; (b) were currently working in specialized gastric cancer wards or gastrointestinal surgical units heavily dedicated to gastric cancer care; (c) had at least 1 year of total clinical experience and at least 6 months of continuous work in their current unit; and (d) provided informed electronic consent. To ensure the specificity of the sample regarding gastric cancer care, nurses working in general surgery, general oncology units were strictly excluded, as were those on internship, in purely administrative roles, or on long-term leave.

A convenience sampling strategy was adopted. The survey was hosted on the online platform “Wenjuanxing” (Changsha Ranxing Information Technology Co., Ltd., China). The survey link was distributed via internal hospital messaging networks (e.g., WeChat workgroups) with the assistance of nursing managers in the target units. Because the anonymous survey link was disseminated through unit managers across multiple hospitals, the exact number of participating institutions, the denominator of eligible nurses who received the invitation, and a formal response rate could not be determined. To ensure data quality and minimize selection bias, settings were configured to allow each IP address and device to submit the questionnaire only once.

### 2.3 Sample size justification

The target sample size was determined based on the number of items in the study instruments, following the widely accepted rule of thumb (Kendall’s sample size estimation method) which suggests a subject-to-item ratio of at least 10:1 to ensure statistical stability (15). The survey instrument comprised the Professional Quality of Life Scale (ProQOL, 30 items), the Connor-Davidson Resilience Scale (CD-RISC, 25 items), and a sociodemographic section (approx. 10 items), totaling 65 items. Consequently, a minimum sample size of 650 participants was calculated (65 items×10).

Taking into account a potential invalid response rate of 10–15% (e.g., short completion time or logical inconsistencies), we aimed to recruit approximately 750 participants. Ultimately, 678 gastric cancer nurses provided valid and complete data. This final sample size not only satisfies the item-ratio requirement but also meets the recommended threshold (*N* > 500) for conducting Latent Profile Analysis (LPA) to ensure accurate class enumeration and stable parameter estimation (16).

### 2.4 Measures

#### 2.4.1 Socio demographic and work related characteristics

A structured self report form was used to collect age, gender, marital status, highest educational level, professional title, hospital level, current department, total years of nursing experience, years working in a gastric cancer related unit, night shift status (yes or no), number of night shifts in the last month, average weekly working hours, history of receiving psychological related training (yes or no) and monthly income. In this study, psychological training referred to self-reported participation in hospital-organized activities related to stress management, emotional regulation, or coping support and was recorded as a yes/no variable. Similar demographic and occupational variables have been employed in previous large scale resilience and burnout surveys among Chinese nurses (14, 17).

#### 2.4.2 Resilience

Psychological resilience was assessed using the 25 item Connor Davidson Resilience Scale (CD RISC), which evaluates the ability to cope with adversity and bounce back from stress across multiple domains such as tenacity, control and spiritual influences (12964174). Each item is rated on a 0 to 4 Likert scale, with higher scores indicating greater resilience; total scores range from 0 to 100. The Chinese version of the CD RISC has demonstrated good internal consistency, convergent validity and factor structure in community and clinical samples (28279394). Studies among Chinese hospital nurses have further confirmed its applicability and reliability in this population (14). In the present study, the total CD RISC score was used as a continuous indicator of resilience.

#### 2.4.3 Compassion fatigue and compassion satisfaction

Compassion fatigue and compassion satisfaction were measured using the Professional Quality of Life Scale, version 5 (ProQOL 5). The ProQOL comprises 30 items forming three 10 item subscales: Compassion Satisfaction, Burnout and Secondary Traumatic Stress. Each item is scored from 1 (never) to 5 (very often), yielding subscale scores ranging from 10 to 50, with higher scores indicating higher levels of the respective construct (31265178). The ProQOL has been widely applied among nurses and other helping professionals to assess compassion fatigue and related constructs (2). Chinese studies have supported the internal consistency and construct validity of the three subscales among clinical nurses (14).

In this study, the total scores of Compassion Satisfaction, Burnout and Secondary Traumatic Stress were used as continuous manifest indicators for the LPA, consistent with prior person centred work on professional quality of life in nursing populations (2).

#### 2.4.4 Occupational outcome variables

Three single item self rating measures were employed to capture key occupational outcomes consistent with previous workforce studies in Chinese nursing (17). Turnover intention was assessed by a single 6 point item asking participants to rate the strength of their intention to leave their current position, with response options ranging from 1 (very low) to 6 (very high); higher scores indicate stronger turnover intention. Brief single item turnover intention measures have shown satisfactory predictive validity for actual turnover and correlate strongly with multi item scales in health care settings (17).

Job satisfaction was measured by a single numerical rating scale ranging from 0 (completely dissatisfied) to 10 (completely satisfied). Similarly, work engagement was measured using a single 0 to 10 rating of overall work engagement, where higher scores reflect higher perceived engagement. Single item global ratings of job satisfaction and engagement have been demonstrated to be psychometrically acceptable and practical in large scale occupational health surveys (2, 17). In this study, both variables were treated as continuous outcomes in profile comparisons.

### 2.5 Data collection procedure

Data were collected between [Month Year] and [Month Year] via an encrypted online survey platform. After receiving an invitation message, eligible nurses accessed the questionnaire through a secure link. The first page provided an information sheet explaining the study purpose, procedures, voluntary nature of participation and data confidentiality. Participants indicated informed consent electronically before proceeding. To minimize careless responding, the platform required completion of all items on the main scales, and logic checks were set for implausible responses (for example, extremely short completion times). Questionnaires failing quality control checks were excluded automatically.

### 2.6 Statistical analysis

Data were analysed using Mplus 8.3 and SPSS 26.0. First, descriptive statistics were calculated for all variables. Pearson correlation coefficients were computed to examine bivariate associations among compassion satisfaction, burnout, secondary traumatic stress and resilience.

Latent profile analysis was then performed to identify subgroups, defined here as groups of nurses with similar patterns of ProQOL scores. Models with one to five profiles were estimated using robust maximum likelihood. Model selection was based on a combination of information criteria (Akaike Information Criterion [AIC], Bayesian Information Criterion [BIC], and sample-size adjusted BIC [aBIC]), entropy, Lo Mendell Rubin adjusted likelihood ratio test and bootstrapped likelihood ratio test, following recommended practices for LPA in psychological research (16). Lower AIC, BIC, and aBIC values indicate better relative model fit. Profile solutions were required to have adequate statistical fit, high classification accuracy (entropy >0.80) and clinically interpretable class sizes, with very small classes (<5 percent of the sample) considered potentially unstable (16).

After selecting the optimal profile solution, differences in ProQOL indicators and occupational outcome variables (turnover intention, job satisfaction, work engagement) across profiles were examined using analysis of variance with *post hoc* comparisons. Finally, multinomial logistic regression was used to explore predictors of profile membership, with the Distressed profile as the reference category. Predictors entered into the final model included age, night shift frequency, weekly work hours, psychological training, and resilience. Educational level and monthly income were collected descriptively but were not included in the final multinomial model. The events per variable ratio satisfied commonly recommended thresholds for multinomial logistic regression (18). For interpretability, the odds ratio for resilience was presented per 10-point increase in CD-RISC score. Results are reported as odds ratios with 95 percent confidence intervals. Statistical significance was set at *p* < 0.05 (two tailed).

### 2.7 Ethical considerations

The study protocol was reviewed and approved by the ethics committee of the coordinating hospital (approval number: (2025)216). Participation was completely voluntary, and anonymity was assured. No identifying information was collected, and data were stored on password protected computers accessible only to the research team. The procedures were consistent with ethical principles for research involving human participants and with prior large scale nursing surveys conducted in China (14, 17).

## 3 Results

### 3.1 Demographic characteristics of participants and basic correlations of variables

A total of 678 gastric cancer nurses were included in the final analysis, constituting a specialized nursing cohort recruited from tertiary hospitals (Table 1). The mean age of the sample was 35.12 years (SD = 7.45), and the vast majority were female (93.8%). Notably, more than half of the sample (55.5%) held a bachelor’s degree, and another 9.3% held a master’s degree or above, reflecting a high level of specialized education in the gastric cancer nursing workforce. However, the data also revealed severe occupational environmental challenges faced by this group: up to 48.4% of nurses undertook 6 or more night shifts in the past month, with 12.3% exceeding 10 shifts, indicating an extremely high physical and cognitive load. In sharp contrast, the professional support system appeared relatively lagging, with only 20.1% of respondents stating they had received systematic psychological training. This structural contradiction of high load and low support provided a potential breeding ground for compassion fatigue. Before proceeding to complex latent variable modeling, we first examined the distribution and interrelationships of major continuous variables (Table 2). Descriptive statistics of the Professional Quality of Life (ProQOL) scale showed that the average score for burnout among gastric cancer nurses was 29.45 (SD = 6.12), and for secondary traumatic stress was 28.14 (SD = 6.35); both were at moderately high levels, suggesting that emotional exhaustion is not an isolated case but a widespread phenomenon in this group. As the core protective resource focused on in this study, the mean score for resilience (CD-RISC) was 72.35 (SD = 14.20), showing considerable inter-individual variation. The results of the Pearson correlation matrix strongly supported the theoretical hypotheses of this study: resilience showed strong negative correlations with burnout (*r* = −0.68, *p* < 0.01) and secondary traumatic stress (*r* = −0.54, *p* < 0.01), implying that individuals with high psychological resilience can more effectively buffer the impact of negative emotions when facing clinical pressure. Meanwhile, resilience was significantly positively correlated with compassion satisfaction (*r* = 0.59, *p* < 0.01), preliminarily indicating that resilience may be a key psychological capital for maintaining professional accomplishment and nursing enthusiasm.

**Table 1:** Socio-demographic and work-related characteristics of the gastric cancer nurses (*N* = 678).

Characteristic	Category	*N*	%
Total		678	100
Age (years)	Mean ± SD	35.12	7.45
Gender	Female	636	93.8
	Male	42	6.2
Marital status	Married	486	71.7
	Unmarried/Other	192	28.3
Education level	Bachelor’s degree or below	615	90.7
	Master’s degree or above	63	9.3
Professional title	Junior Nurse	361	53.2
	Senior Nurse/Supervisor	317	46.8
Night shifts (last month)	≤5 shifts	350	51.6
	6–10 shifts	245	36.1
	>10 shifts	83	12.3
Weekly work hours	Mean ± SD	44.52	5.3
Psychological training	No	542	79.9
	Yes	136	20.1
Monthly income (CNY)	Mean ± SD	10,850	2,400

**Table 2:** Means, standard deviations, and Pearson correlations among key study variables.

Variables	Mean (SD)	1	2	3	4
1. Compassion satisfaction (CS)	33.20 (5.88)	1			
2. Burnout (BO)	29.45 (6.12)	−0.60**	1		
3. Sec. Traumatic stress (STS)	28.14 (6.35)	−0.32**	0.61**	1	
4. Resilience (CD-RISC)	72.35 (14.20)	0.59**	−0.68**	−0.54**	1

CS, compassion satisfaction; BO, burnout; STS, secondary traumatic stress; CD-RISC, Connor-Davidson Resilience Scale. **p < 0.01.

### 3.2 Identification and model fit of latent profiles of compassion fatigue among gastric cancer nurses

To identify whether nurses clustered into distinct patterns of professional quality of life rather than relying only on sample-average scores, this study adopted a person-centered Latent Profile Analysis (LPA) approach. We utilized burnout, secondary traumatic stress, and compassion satisfaction as manifest indicators and systematically fitted latent class models ranging from 1 to 5 subgroups (Table 3). As the number of profiles increased, the AIC, BIC, and aBIC decreased, with lower values indicating better relative fit. However, determining the optimal model requires a comprehensive consideration of statistical significance, classification precision, and the parsimony and interpretability of the results. The analysis results showed that the 3-profile model demonstrated the optimal comprehensive performance. First, the Entropy value of this model was as high as 0.89, indicating extremely high classification clarity and high certainty in distinguishing individual class membership. Second, the Lo–Mendell–Rubin likelihood ratio test (LMR) and Bootstrapped Likelihood Ratio Test (BLRT) both reached highly significant levels (*p* < 0.01) in the 3-profile model, providing strong statistical evidence that the 3-profile solution was significantly superior to the 2-profile solution. Although the 4-profile model showed slight optimization in some information criteria, its LMR test result was no longer significant (*p* = 0.254), and the separated fourth class had a sample size that was too small (<5%), which lacks practical guidance significance in clinical management and easily leads to unstable statistical results. Therefore, based on the dual consideration of statistical goodness-of-fit and theoretical interpretability, this study finally determined the 3-profile model as the optimal solution.

**Table 3:** Model fit indices for latent profile analysis of compassion fatigue (1–5 profiles).

Model	AIC	BIC	aBIC	Entropy	LMR (*p*)	BLRT (*p*)	Class proportions (%)
1-Profile	12560.4	12590.2	12570.1	–	–	–	100%
2-Profile	11890.5	11940.8	11910.3	0.82	<0.001	<0.001	42%/58%
3-Profile	11420.2	11490.5	11440.6	0.89	0.003	<0.001	22%/48%/30%
4-Profile	11400.1	11510.9	11435.2	0.84	0.254	0.045	15%/20%/35%/30%
5-Profile	11390.8	11540.3	11442.1	0.81	0.41	0.12	

AIC, Akaike Information Criterion; BIC, Bayesian Information Criterion; aBIC, sample-size adjusted Bayesian Information Criterion; LMR, Lo–Mendell–Rubin adjusted likelihood ratio test; BLRT, bootstrapped likelihood ratio test. Lower AIC, BIC, and aBIC values indicate better relative model fit.

### 3.3 Characterization and naming of latent profiles

**Figure 1:**
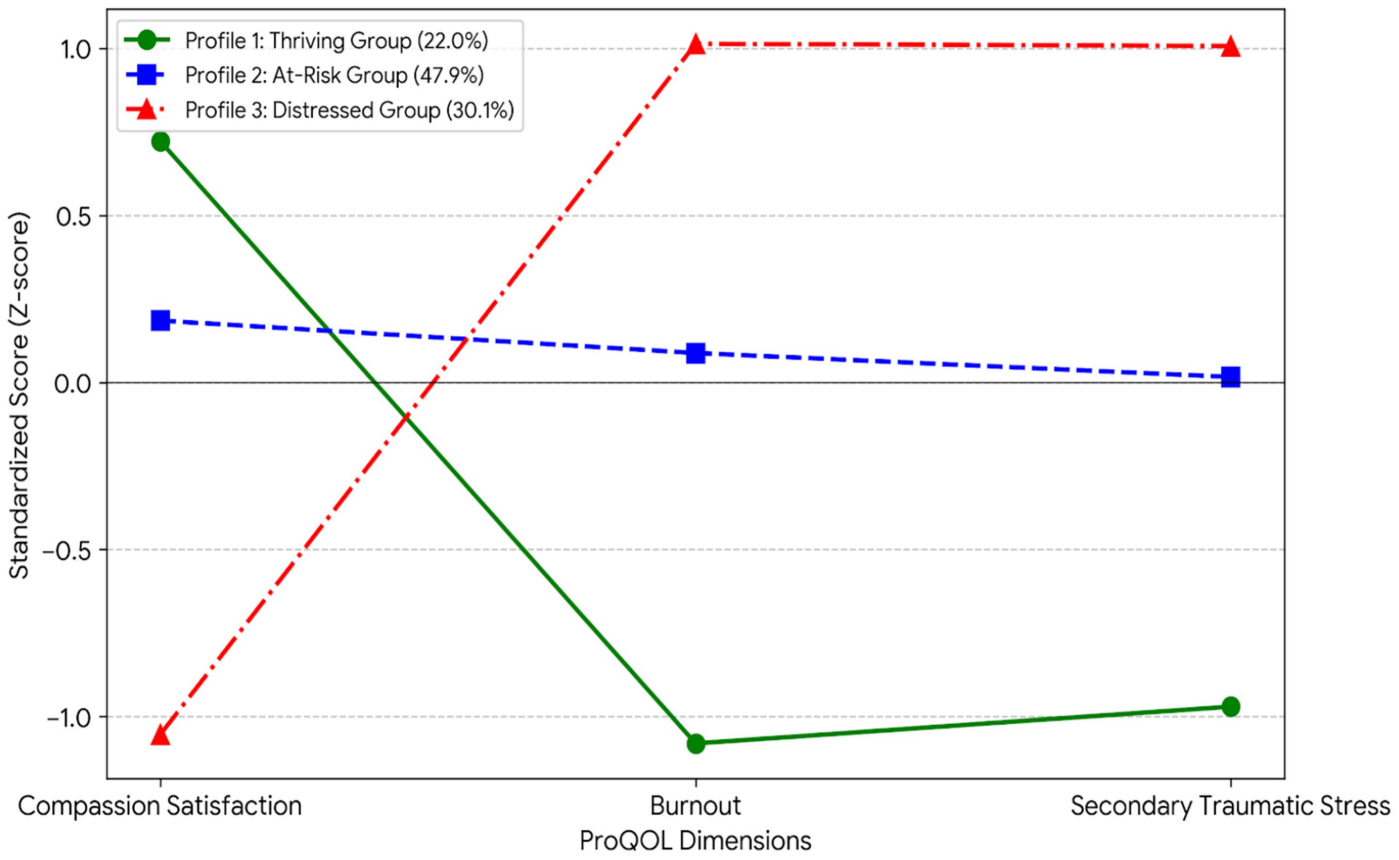
Latent profiles of compassion fatigue among gastric cancer nurses.

Based on the optimal 3-profile model, we identified three nurse subgroups with distinct psychological characteristics (Table 4). The standardized score patterns of each profile on the three ProQOL dimensions were plotted to visually display the morphological differences between groups (Figure 1). The first class of nurses accounted for 22.0% of the total sample (*n* = 149), with characteristics manifesting as an ideal adaptive pattern of high satisfaction-low depletion, that is, compassion satisfaction was significantly higher than the sample mean (42.34 ± 3.51), while burnout (19.80 ± 4.20) and secondary traumatic stress (18.50 ± 4.10) scores were at the lowest levels. Considering that this group maintained an excellent professional state despite the high-intensity pressure of gastric cancer care, we named it the Thriving Profile. The second class was the largest group in the sample, reaching 47.9% (*n* = 325), approaching half. This group showed moderate levels in compassion satisfaction, burnout, and traumatic stress. Although they did not yet exhibit extreme pathological symptoms, their burnout level (28.40 ± 3.90) was notably higher than that of the Thriving Profile, placing them in a gray zone of mental health; thus, we named it the At-Risk Profile. The third class accounted for approximately one-third of the total sample (30.1%, *n* = 204). Their characteristics presented a typical crisis pattern of “low satisfaction-high depletion,” characterized by extremely low compassion satisfaction (21.65 ± 3.89), accompanied by extremely high levels of burnout (38.90 ± 4.50) and secondary traumatic stress (36.80 ± 5.20). This group represents a high-incidence population for professional burnout facing a severe psychological crisis; therefore, we named it the Distressed Profile. ANOVA results confirmed that the differences among these three profiles on all clustering indicators had extremely high statistical significance (*F* > 800, *p* < 0.001), fully supporting the discriminant validity between profiles.

**Table 4:** Comparison of Professional Quality of Life (ProQOL) dimensions across the three identified latent profiles.

ProQOL dimensions	Profile 1 “Thriving” (*n* = 149, 22.0%)	Profile 2 “At-Risk” (*n* = 325, 47.9%)	Profile 3 “Distressed” (*n* = 204, 30.1%)	*F*	*p*	*Post-hoc*
Compassion Satisfaction	42.34 ± 3.51	32.50 ± 4.10	21.65 ± 3.89	1145.21	<0.001	1 > 2 > 3
Burnout	19.80 ± 4.20	28.40 ± 3.90	38.90 ± 4.50	985.6	<0.001	3 > 2 > 1
Sec. Traumatic Stress	18.50 ± 4.10	27.20 ± 4.60	36.80 ± 5.20	820.3	<0.001	3 > 2 > 1

ProQOL, Professional Quality of Life. Values are mean ± SD. *Post-hoc* indicates the direction of pairwise differences between profiles.

### 3.4 External validity validation of latent profiles: association with occupational outcomes

To verify whether the above classification has actual clinical predictive value, we further examined the relationship between different psychological profile memberships and key nurse occupational outcome variables (turnover intention, job satisfaction, work engagement) (Table 5). The analysis results revealed a significant gradient effect, meaning that nurses’ internal psychological profile categories showed high consistency with their external occupational attitudes. The most concerning finding came from the Distressed Profile group. This group demonstrated an alarming turnover tendency, with an average turnover intention score as high as 4.98 (out of 6), and further frequency analysis showed that up to 80.4% of members in this group reported high turnover intention. In contrast, the turnover intention of the Thriving Profile nurses was extremely low (1.85), with only 6.0% having thoughts of leaving, while the At-Risk Profile nurses fell between the two (3.12). Regarding job satisfaction and work engagement, the same gradient pattern persisted: Thriving Profile nurses scored the highest, showing extremely high work enthusiasm and organizational commitment; whereas Distressed Profile nurses scored the lowest, showing obvious work alienation. Chi-square tests and ANOVA both showed that differences between groups reached highly significant levels (*p* < 0.001). This result strongly suggests that the latent profiles identified in this study not only reflect the mental health status of nurses but are also potent indicators predicting their retention behavior and work performance. Identifying Distressed Profile nurses has important managerial significance for preventing talent loss.

**Table 5:** Differences in turnover intention, job satisfaction, and work engagement across the three latent profiles.

Outcome variables	Profile 1 (Thriving)	Profile 2 (At-Risk)	Profile 3 (Distressed)	*χ*^2^/*F*	*p*
Turnover intention (Score 1–6)	1.85 ± 0.82	3.12 ± 0.95	4.98 ± 1.10	450.25	<0.001
Job satisfaction (Score 0–10)	8.90 ± 1.20	6.50 ± 1.50	3.20 ± 1.40	560.1	<0.001
Work engagement (Score 0–10)	8.50 ± 1.30	6.80 ± 1.40	4.10 ± 1.60	320.4	<0.001
Leave intention (Binary)					
No intention	140 (94.0%)	200 (61.5%)	40 (19.6%)	189.5	<0.001
High intention	9 (6.0%)	125 (38.5%)	164 (80.4%)		

Values are mean ± SD or *n* (%), as appropriate. *χ*^2^ was used for the binary comparison and F was used for continuous outcomes.

### 3.5 Predictors of profile membership and associations with resilience

To examine factors associated with membership in different psychological profiles, we constructed a multinomial logistic regression model using the Distressed Profile (Profile 3) with the worst mental health status as the reference group. The final model included age, night shift frequency, weekly work hours, psychological training, and resilience as predictor variables, aiming to calculate the Odds Ratio (OR) of nurses belonging to the healthier Thriving Profile or At-Risk Profile (Table 6). When resilience was expressed per 10-point increase in CD-RISC score for interpretability, higher resilience was associated with greater odds of belonging to the Thriving Profile rather than the Distressed Profile (OR = 5.23, 95% CI: 3.11–9.31, *p* < 0.001) and of belonging to the At-Risk Profile rather than the Distressed Profile (OR = 2.37, 95% CI: 1.63–3.71, *p* < 0.001). This pattern supports an association between higher resilience and more favorable profile membership. Besides individual psychological traits, work environment factors also played a critical role. Night shift frequency was also associated with profile membership. Compared with nurses who had fewer than 5 night shifts per month, the likelihood of nurses with high-frequency night shifts (>5/month) belonging to the Thriving Profile was drastically reduced by 75% (OR = 0.25, 95% CI: 0.12–0.52, *p* < 0.001). Receipt of psychological training was associated with increased odds of Thriving membership (OR = 2.10, 95% CI: 1.20–3.65, *p* = 0.009). This finding provides a clear direction for intervention for hospital administrators: optimizing shift scheduling to reduce extreme night shift loads and providing systematic psychological skills training are effective pathways to improve the professional quality of life of gastric cancer nurses and reduce the risk of compassion fatigue.

**Table 6:** Multinomial logistic regression analysis predicting latent profile membership with resilience and selected factors.

Predictors	Profile 1 (thriving) vs. Profile 3 (distressed) OR (95% CI)	*p*	Profile 2 (at-risk) vs. Profile 3 (distressed) OR (95% CI)	*p*
(Intercept)	0.45		0.85	
Age	1.02 (0.98–1.06)	0.35	1.01 (0.98–1.04)	0.62
Night Shifts (>5/month)	0.25 (0.12–0.52)	<0.001	0.65 (0.40–1.05)	0.08
Weekly Work Hours	0.88 (0.82–0.94)	<0.001	0.95 (0.90–1.01)	0.095
Psychological Training (Yes)	2.10 (1.20–3.65)	0.009	1.45 (0.90–2.35)	0.12
Resilience (per 10-point increase in CD-RISC)	5.23 (3.11–9.31)	<0.001	2.37 (1.63–3.71)	<0.001

OR, odds ratio; CI, confidence interval; CD-RISC, Connor-Davidson Resilience Scale. The Distressed profile was the reference category. Resilience is presented per 10-point increase in CD-RISC score.

## 4 Discussion

This study identified three distinct latent profiles of professional quality of life among nurses caring for patients with gastric cancer and showed that these configurations are strongly linked to key occupational outcomes. The overall pattern of moderately high burnout and secondary traumatic stress with only moderate compassion satisfaction is similar to findings in large samples of Chinese tertiary hospital nurses and other inpatient settings, where compassion fatigue has been described as a prevalent and systemic risk rather than an exceptional condition (19, 20). Within this high pressure context, the latent profile approach adds value by moving beyond average scores and revealing heterogeneous psychological subgroups that would otherwise be obscured.

The Thriving profile, marked by high compassion satisfaction and low burnout and secondary traumatic stress, closely resembles high professional quality of life or balanced protection profiles reported among palliative care professionals and general nursing staff (21, 22). Nurses in this class demonstrated very low turnover intention and high work engagement, supporting the notion that compassion satisfaction functions as a positive psychological resource that sustains motivation and commitment despite chronic exposure to suffering (23). In contrast, the Distressed profile showed the classical crisis pattern of low satisfaction combined with very high depletion and was strongly associated with elevated turnover intention. Similar high risk clusters have been observed among oncology nurses, who often report low compassion satisfaction but high burnout and compassion fatigue in relation to cumulative exposure to cancer related loss and complex treatments (19, 24). The present findings therefore extend existing evidence by documenting a sizable psychologically endangered subgroup within a narrowly defined gastric cancer specialist workforce. Compared with broader oncology samples, gastric cancer nurses may experience a distinctive combination of perioperative management, nutritional support, and sustained contact with patients and families, which may make profile-specific support particularly relevant in this setting (1, 2). The clear gradient across profiles in turnover intention, job satisfaction, and work engagement provides strong external validation of the latent classification. Longitudinal work in other nurse populations has shown that less favorable professional quality of life profiles predict later increases in turnover intention and decreases in life satisfaction, partly via reduced job satisfaction and work engagement (22). Although the present study is cross sectional, the same monotonic pattern suggests that these profiles capture clinically meaningful risk strata rather than arbitrary statistical groupings. In practical terms, the Distressed profile appears to identify nurses who are not only suffering psychologically but also most likely to disengage from their roles or leave the organization, which has direct implications for workforce stability in oncology care. Resilience was strongly associated with more favorable profile membership within this framework. Higher resilience was strongly associated with lower burnout and secondary traumatic stress and higher compassion satisfaction, consistent with studies in mental health and general hospital nurses where resilience correlates positively with professional quality of life and negatively with indicators of compassion fatigue (9, 25). When interpreted per 10-point increase in CD-RISC score, the association with Thriving rather than Distressed membership was more clinically interpretable. These findings align with evidence that resilience can partially mediate the relationship between compassion fatigue and outcomes such as job satisfaction, turnover intention, and perceived quality of care (26). Together, the results suggest that resilience may be an important psychological resource linked to more adaptive professional quality-of-life patterns.

Importantly, the present data indicate that resilience should not be regarded as a purely fixed trait. Nurses who had received systematic psychological training showed higher odds of belonging to the Thriving profile even after adjustment for the variables retained in the final model. This observation converges with evaluations of compassion fatigue resiliency programs and related educational interventions, which, despite mixed effects on burnout and secondary traumatic stress, have demonstrated improvements in compassion satisfaction, awareness, and coping skills among oncology nurses (27–29). Such findings support a combined approach in which resilience is conceptualized as both an internal disposition and a set of learnable skills that can be strengthened through structured programs. And this study underscores that individual psychological resources operate within a demanding structural environment. High night shift frequency emerged as a strong negative predictor of Thriving membership, consistent with growing evidence that shift work disorder, long or frequently rotating night shifts, and inadequate recovery are associated with increased burnout, mental health problems, and fatigue among nurses (30–32). Excessive night shifts may be linked to poorer sleep quality, cognitive functioning, and emotional regulation, which could in turn be associated with lower resilience and less favorable profile membership. These results reinforce calls for organizational interventions in staffing and scheduling, indicating that resilience building alone is unlikely to succeed if systemic workload pressures remain unaddressed.

Several practice implications follow. First, routine assessment using ProQOL based indicators could be employed to classify nurses into latent risk profiles and to flag Distressed staff for early, possibly multidisciplinary, support. Second, resilience focused psychological training and psychoeducation about compassion fatigue should be incorporated into orientation and ongoing professional development for gastric cancer nurses, with tailored emphases for Thriving, At Risk, and Distressed groups. Third, hospital administrators should review night shift arrangements and total workload, as reducing extreme night shift burden appears critical for preserving both resilience and professional quality of life. Finally, unit leaders could integrate profile aware supervision, for example by providing mentoring and leadership opportunities to Thriving nurses while offering targeted coaching and mental health referral pathways to Distressed nurses.

This study has limitations. The cross sectional design precludes causal inference regarding the directionality between resilience, profile membership, and occupational outcomes, and bidirectional relationships are plausible. All measures were self reported and collected at a single time point, which may introduce common method variance and social desirability bias; a formal statistical test of common method bias was not conducted. The sample consisted solely of gastric cancer nurses within a particular health care system, which may limit generalizability to other oncology subspecialties or countries. In addition, turnover intention, job satisfaction, and work engagement were assessed using single-item measures, which are practical in workforce surveys but less precise than multi-item scales. Because the anonymous online recruitment procedure did not capture the denominator of invited nurses, a formal response rate could not be calculated. Although entropy supported good classification, average posterior probabilities and additional sensitivity analyses were not reported. In addition, potential organizational determinants such as staffing ratios, leadership style, and institutional culture were not directly measured and could partly explain profile differences.

Future research should employ longitudinal designs to track transitions between profiles and to determine whether changes in resilience or work conditions precede shifts in professional quality of life. Experimental and quasi experimental studies are needed to test whether resilience enhancing programs and scheduling reforms can move nurses from Distressed or At Risk into Thriving profiles and whether such transitions yield sustained improvements in retention, mental health, and patient outcomes. Incorporating objective indicators, such as sickness absence, incident reports, and patient satisfaction, alongside self reported professional quality of life would provide a more comprehensive picture of the practical impact of psychological configurations. Despite these limitations, the present study contributes to a growing body of evidence that resilience and modifiable work environment factors jointly shape the professional quality of life of oncology related nurses and offers concrete targets for multilevel interventions to safeguard this highly specialized workforce.

## Data Availability

The original contributions presented in the study are included in the article/supplementary material, further inquiries can be directed to the corresponding author/s.
